# Genital HSV Detection among HIV-1-Infected Pregnant Women in Labor

**DOI:** 10.1155/2011/157680

**Published:** 2011-03-27

**Authors:** Janna Patterson, Jane Hitti, Stacy Selke, Meei-Li Huang, D. Heather Watts, Zane Brown, Lawrence Corey, Anna Wald

**Affiliations:** ^1^Division of Neonatology, Department of Pediatrics, University of Washington, P.O. Box 356320, Seattle, WA 98195-6320, USA; ^2^Department of Obstetrics and Gynecology, University of Washington, Seattle, WA 98195-6320, USA; ^3^Department of Laboratory Medicine, University of Washington, Seattle, WA 98195-6320, USA; ^4^Vaccine and Infectious Disease Division, Fred Hutchinson Cancer Research Center, Seattle, WA 98109, USA; ^5^Pediatric, Adolescent and Maternal AIDS Branch, Eunice Kennedy Shriver National Institute of Child Health and Human Development, Rockville, MD 20847, USA; ^6^Department of Medicine, University of Washington, Seattle, WA 98195-6320, USA; ^7^Department of Epidemiology, University of Washington, Seattle, WA 98195-6320, USA

## Abstract

*Objective*. To compare genital HSV shedding among HIV-positive and HIV-negative women. *Methods*. Women with and without known HIV infection who delivered at the University of Washington Medical Center between 1989–1996 had HSV serologies done as part of clinical care. Genital swabs from HSV-2-seropositive women were evaluated by real-time quantitative HSV DNA PCR. *Results*. HSV-2 seroprevalence was 71% and 30% among 75 HIV-positive and 3051 HIV-negative women, respectively, (*P* < .001). HSV was detected at delivery in the genital tract of 30.8% of HIV-seropositive versus 9.5% of HIV-negative women (RR = 3.2, 95% CI 1.6 to 6.5, *P* = .001). The number of virion copies shed per mL was similar (log 3.54 for HIV positive versus 3.90 for HIV negative, *P* = .99). *Conclusions*. Our study demonstrated that HIV-, HSV-2-coinfected women are more likely to shed HSV at delivery.

## 1. Introduction

The rate of infant HIV infection in the USA has plummeted with the advent of routine HIV testing during pregnancy and the availability of potent antiretroviral therapy. These public health advances shift focus to prevention of other comorbid conditions in HIV-infected women and their infants. 

Herpes simplex virus type 2 infections are prevalent among women and among persons with HIV infection. In resource-limited settings with high HIV prevalence such as the Central African Republic, HSV-2 antibody prevalence among HIV-infected women is 91% [1]. Studies have reported an increased risk of HIV infection in infants born to HSV-2- and HIV-coinfected women or women who have clinical genital herpes during pregnancy [2, 3]. The risk is likely mediated by an increase in plasma and/or mucosal HIV RNA during HSV reactivation [4] and may be abrogated by antiretroviral therapy. Less attention has been given to the potential for increased risk of neonatal herpes in infants of coinfected women. Persons with HIV and HSV-2 have increased rates of genital HSV reactivation, and, if they have advanced immunosuppression, have a higher rate of mucosal HSV shedding [1, 5–7]. Because HSV genital shedding during labor is the strongest risk factor for neonatal herpes (relative risk > 300) [8], such HIV- and HSV-2-infected women could be at increased risk for transmitting HSV to their newborns. Neonatal herpes is a devastating disease for infants with a 30% mortality rate in the case of adequately treated disseminated disease [9]. 

To assess the potential for HSV transmission to the neonate among HIV-infected women, we compared rates of HSV-2 infection and genital HSV shedding among HIV-infected and HIV-negative women.

## 2. Methods

Women with known HIV infection who delivered at the University of Washington Medical Center (UWMC), a tertiary care referral center, between 1989 and 1996 were included in the study. Additionally, women without known HIV infection delivering at UWMC between 1995-96 were included. All women were receiving comprehensive prenatal care at UWMC or affiliated clinics. During the study period, type-specific HSV serologies were included in routine prenatal care at our institution as part of a large, continuing study of HSV in pregnancy approved by the University of Washington Institutional Review Board [8]. Genital swabs were obtained in a subset of women expected to deliver vaginally as part of an ongoing study of infant exposure to HSV during birth [8, 10, 11]. Genital secretions were obtained from the labia majora and minora as well as perianal and periclitoral areas with a dacron-tipped swab. A second swab was collected from the ectocervix, endocervix, and posterior vaginal fornices during a speculum exam. HSV was cultured from the genital secretions swab, as previously reported [12]. The remaining sample was stored frozen for later testing by PCR.

HIV serologic testing was performed using a commercial enzyme-linked immunosorbent assay (Genetic Systems HIV-1/HIV-2 Plus O EIA, Bio-Rad, Redmond, WA) with Western blot confirmation for samples testing positive. Type-specific HSV serostatus was determined with the University of Washington Western Blot [13]. Genital swabs were evaluated by real-time quantitative HSV DNA polymerase chain reaction (PCR) assay [14].

The prevalence of HSV-1 and HSV-2 antibodies and the median and range of log copies of HSV shed were compared among women with and without HIV infection. Categorical variables were compared using chi-square or Fisher's exact test, and continuous variables were compared using the Mann-Whitney test. Risk ratios with 95% confidence intervals (CIs) were also calculated for the difference between these groups. A 2-sided *P* value of  .05 or less was considered statistically significant. Statistical analyses were conducted with SPSS for Windows, version 11.5.

## 3. Results

During the study interval, 75 known HIV seropositive women had a total of 85 deliveries included in this analysis. During 1995-1996, there were 3099 consecutive women admitted to Labor and Delivery who were not known to be HIV infected, and 3051 (98%) of these women had HSV serology testing available; these were included as the comparator group. The median age was 27 (range 17, 37) for HIV-seropositive women and 29 (range 15, 49) for HIV-negative women (*P* = .6). Among HIV-seropositive women, 50% were white, 21% black, and 29% other races. Among HIV seronegative women, 46% were white, 34% black, and 20% were other races. Nulliparous women comprised 31% of HIV-seropositive and 34% of HIV-seronegative women. 

Among 75 HIV-seropositive women, 71.2% had antibody to HSV-2 (28.7% to HSV-2 only and 42.5% to HSV-2 and HSV-1), 21.9% to HSV-1 only, and 6.8% were HSV seronegative. In contrast, among 3051 HIV-seronegative women, the HSV-2 seroprevalence rate was 30.3% (11.1% seropositive for HSV-2 only and 19.2% seropositive for both HSV-1 and HSV-2), while 50.6% were HSV-1 seropositive only and 19.1% were HSV seronegative (*P* < .001 for the difference in frequency of HSV-2 antibody between HIV seropositive and HIV seronegative women).

In a subset of HSV-2 seropositive women in the study, the swab of genital secretions obtained at the time of delivery was evaluated with HSV DNA PCR swabs. Among 26 HIV-positive, HSV-2-seropositive women who were evaluated, none had genital lesions, and all had swabs obtained for HSV from genital secretions. Among 635 HIV-negative, HSV-2-seropositive women who were evaluated, 13 had lesions at delivery, and a swab for HSV was obtained from the remaining 622 for HSV detection. Vaginal deliveries were common in both groups of HSV-2-infected women without lesions as 79% of HIV-positive women and 96% of HIV-negative women delivered vaginally. 

**Figure 1:**
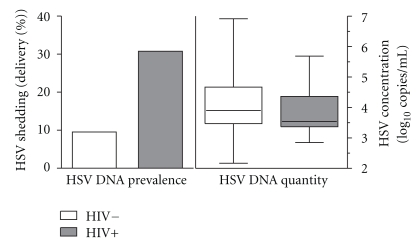
HSV shedding by PCR in HIV-positive and HIV-negative women at delivery.

Genital HSV was detected in 8 (30.8%) of 26 HIV-, HSV-2-infected women at the time of delivery. Seven of these women were HSV-2 PCR positive only, and one was HSV-2 and HSV-1 positive by PCR swab. Specifically, HSV DNA was detected in all 8 vulvar swabs, and in 4 of the 8 HSV was also detected in the cervical swabs. Among the 622 HIV seronegative women who had swabs taken for PCR, 59 (9.5%) women had HSV detected. Of 59 women who had HSV detected, 27 were positive both at vulva and cervix, 24 only at the vulva, and 6 only at the cervix; an additional 2 women were positive at the cervix but vulvar swabs were not available. The risk of HSV detection among HIV-seropositive women was 3.2-fold higher (95% CI 1.6 to 6.5, *P* = .001) than among HIV-seronegative women (Table 1). Quantitative amount of HSV in the swabs did not differ significantly between HIV-seropositive and HIV-seronegative women with the median number of virion copies per mL of log 3.54 (range 2.85, 5.69) for HIV-positive women versus log 3.90 (range 2.17, 6.92) for HIV-negative women (*P* = .99, Figure 1).

## 4. Discussion

Our study showed that HSV-2- and HIV-coinfected pregnant women were more likely to shed HSV at delivery than their HIV negative counterparts. The quantitative amounts of shedding did not differ between HIV-positive and HIV-negative women, perhaps reflecting the fact that all had established HSV-2 infection. Viral shedding is the strongest risk factor for transmission of HSV from the mother to the neonate at birth [8]. While most transmissions to newborns occur from pregnant women who have recently acquired genital HSV and have not developed a detectable antibody response by the time of delivery, women with established HSV-2 infection are also at risk for transmitting HSV to their neonate, but at a much lower rate [8]. 

Prior studies have addressed the role of HSV-2 in increasing the risk of perinatal HIV transmission [15], especially in Africa where HSV-2 infection affects most HIV-1-seropositive pregnant women [16] and highly active antiretroviral therapy is not universally used during pregnancy. Drake et al. [4] showed that genital ulcers were associated with increased plasma HIV-1 RNA and increased risk of intrapartum transmission of HIV and calculated that 14% of HIV-1 transmissions were attributable to maternal HSV-2 ulcers. The Cowan et al. study in Zimbabwe suggested that 28% of intrapartum HIV transmissions are potentially attributed to prevalent maternal HSV-2 [16]. Also concerning this cohort is the additional 17% (29 of 193) of initially HSV-2-seronegative women who subsequently seroconverted to HSV-2 in the immediate peripartum period. This suggests that a substantial number of women acquire new HSV-2 infection peripartum, an event associated with a 30–50% risk of HSV transmission to the neonate [17]. We are aware of a single-case report of HSV transmission to the neonate from an HIV-infected woman [18]. In this case, the HSV infection was acquired at the end of pregnancy. Of note, this case was observed in a resource-rich setting, underscoring that a diagnostic workup for neonatal HSV is less likely to be routinely performed in the developing world. To our knowledge, no systematic study evaluating the incidence of neonatal HSV in resource-poor areas with high HIV prevalence has been done.

Our study was limited by a relatively small number of HIV-positive pregnant women. In addition, we focused on women who are HSV-2 seropositive, who are at lesser risk for transmitting neonatal herpes than women who acquire genital HSV in late pregnancy. Further studies of the risk of neonatal HSV among HIV-infected women should include women who are at risk for HSV acquisition in the peripartum period. Additionally, while routine heel sticks on infants born to the presumed HIV-negative women during did not reveal any unrecognized HIV infections, many of these women did not have a confirmed negative HIV test, as the study was done prior to routine HIV testing in pregnancy [19].

## 5. Conclusions

While suppressive therapy for HSV is recommended, and utilized, for prevention of genital lesions at labor and cesarean deliveries for women with a clinical history of genital herpes [20], the impact of this approach on neonatal HSV is unknown. The effect of this strategy on the high rate of HSV shedding in labor among HIV-infected, HSV-2-seropositive women has not been studied. In addition, antiviral therapy for HSV is used infrequently in resource-poor countries. Suppressive therapy with acyclovir and valacyclovir has been shown to reduce plasma and genital HIV RNA levels by 0.25–0.50 log but not to impact the risk of sexual transmission of HIV [21–23]. Ongoing studies will evaluate whether valacyclovir can reduce the risk of mother to child HIV transmission during late pregnancy, the intrapartum period, and breastfeeding.

##  Conflict of Interests

The authors declare no conflict of interests.

## Figures and Tables

**Table 1:** HSV PCR positivity in vaginal swabs among HSV-2-seropositive, HIV-positive and HIV-negative women at delivery.

Swab site	HIV positive women	HIV negative women	Risk ratio, 95% CI	*P*-value
Any site	8/26 (30.8%)	59/622 (9.5%)	3.2 (1.6, 6.5)	.001
Cervix	4/25 (16.0%)	35/588 (6.0%)	2.7 (0.9, 7.3)	.053
Vulva	8/24 (33.3%)	51/610 (8.4%)	4.0 (2.0, 8.1)	<.001
Both cervix and vulva	4/23 (17.4%)	27/576 (4.7%)	3.7 (1.3, 10.3)	.012
